# A robust reporting system for measurement of SARS-CoV-2 spike fusion efficiency

**DOI:** 10.1038/s41392-022-01037-4

**Published:** 2022-06-06

**Authors:** Cong Huang, Yang Yang, Peng Yang, Fei Wang, Xinyu Li, Xiang Song, Yiming Wang, Cuiyun Yu, Xuejun Wang, Shengqi Wang

**Affiliations:** 1 Hunan Province Cooperative Innovation Center for Molecular Target New Drug Study, University of South China, 421001 Hengyang, China; 2 State Key Laboratory of Pathogen and Biosecurity, Beijing Institute of Microbiology and Epidemiology, 100850 Beijing, China; 3 Department of Pharmaceutics, Shenyang Pharmaceutical University, 110016 Shenyang, China

**Keywords:** Cytological techniques, Molecular biology

**Dear Editor**,

During the COVID-19 pandemic, several SARS-CoV-2 variants such as Alpha, Delta, and Omicron successively became dominant worldwide. The infection of SARS-CoV-2 is triggered by the binding of spike protein to the cell-surface receptor angiotensin-converting enzyme 2 (ACE2), which then fuses with cell membrane or lysosomal membrane after endocytosis.^1^ Extensive cell fusion and syncytia formation are a signature feature of severe SARS-CoV-2 and may play an important role in COVID-19 pathogenesis. Research has shown that SARS-CoV-2 spike-mediated cell fusion leads to the formation of abnormal and multinucleated cells in the lungs of patients.^2^ Accordingly, the fusion ability of SARS-CoV-2 spike protein may be a leading indicator of viral infectivity and disease severity in SARS-CoV-2 patients. Notably, a multibasic furin cleavage site at the 682-685 residues in spike protein is of vital importance for cell–cell fusion.

Considering the important role of spike fusion in SARS-CoV-2 infection, a robust reporting system was developed herein by fusing a pair of split NanoLuc luciferase and split mNeonGreen protein with a pair of interacting leucine zippers (bFos-bJun) (Fig. 1a). The split proteins must have sufficient freedom of movement in the complex to allow them to collide with one another frequently enough to promote the reformation. Consequently, several fusion proteins denoted as SJNG, NGJS, LFCG, and CGFL were designed to determine the appropriate interaction partners. When the four fusion proteins were transfected separately, neither significant NanoLuc luciferase activity nor mNeonGreen signal was detected (Fig. 1b and supplementary Fig. 1), suggesting that the reporting system had low background signals. When a pair of fusion proteins was co-expressed, the interaction between bFos and bJun restored the NanoLuc luciferase activity and mNeonGreen signal to different extents, and the combination of NGJS and CGFL produced the optimum results (Fig. 1b and supplementary Fig. 1). Thus, NGJS and CGFL were selected as the reporter partner and applied in subsequent assays.

**Fig. 1:**
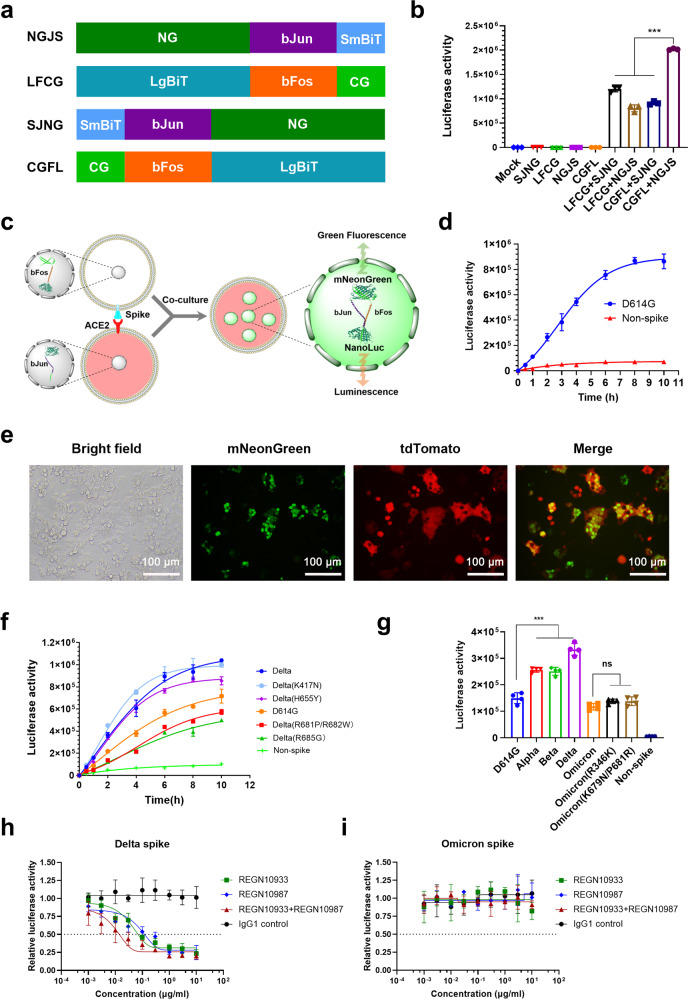
Design and application of the three-functional cell-fusion reporting system. **a** Four different recombining proteins comprising split mNeonGreen and split NanoLuc were fused with different ends of bJun or bFos. NG and CG represent the N- and C-terminal fragments of mNeonGreen, respectively. SmBiT and LgBiT represent the small and large fragments of NanoLuc, respectively. **b** The luciferase activities of these four different recombining proteins were detected after transfecting or co-transfecting their plasmids into HEK293T cells for 36 h. Mock indicated that conditions were consistent, except that no plasmid was transfected. **c** Schematic of this three-functional reporting system for cell-fusion assay. HEK293T cells expressing CGFL and spike were co-cultured with cells expressing NGJS, ACE2, and tdTomato to trigger cell fusion. The green fluorescence signal and luciferase activity were restored and located in the nucleus. **d** The luciferase activities of cell-fusion mediated by the spike D614G variant were measured at the indicated time point post cell co-culture, and plasmids without spike expression served as the negative control (non-spike). **e** The fluorescence signals of the spike D614G mediated cell fusion were observed at 10 h post cell co-culture by using a microscope. Green fluorescence represents recombinant mNeonGreen, and red fluorescence represents cells expressing ACE2. Scale bar: 100 μm. **f** The luciferase activities of Delta spike mutants mediated cell fusion were detected at the indicated time point post cell co-culture. **g** The luciferase activities of typical VOC spikes and Omicron spike mutants mediated cell fusion were detected at 10 h post cell co-culture. **h, i** The relative luciferase activities of Delta and Omicron spike-mediated cell fusion in the presence of neutralizing antibodies were detected at 4 h post cell co-culture.

This reporting system had three functions: (1) after cells fused, the split NanoLuc luciferase recombined and ensured the quantitative detection of cell-fusion efficiency; (2) the split mNeonGreen protein recombined to provide a visual fluorescence signal; and (3) the nuclear location of bFos-bJun enabled intuitive observation of the number of fused cells in the syncytia (Fig. 1c). To verify the three functions of this reporting system, HEK293T cells co-transfected with CGFL and SARS-CoV-2 spike (D614G) plasmids served as effector cells, whereas HEK293T cells co-transfected with NGJS and pLVX-ACE2-IRES-tdTomato plasmids (for ACE2 and tdTomato red fluorescence protein expression) served as target cells. Fusion occurred between these effector and target cells mediated by the interaction of SARS-CoV-2 spike and ACE2 proteins. After fusion, the split NanoLuc and mNeonGreen proteins were reassociated and produced luminescence and green fluorescence (Fig. 1d, e). The number of nuclei (green) in a syncytium (red) represented how many cells fused to one (Fig. 1e). To determine the linearity limit of the NanoLuc luciferase activity, a cell-number titration experiment was performed. Results showed a good linear dynamic range when the NanoLuc luciferase activity was between 1.0 × 10^3^ and 1.0 × 10^6^ (supplementary Fig. 2). Alternatively, the mNeonGreen can be measured dynamically using live cell imagers or microplate reader with fluorescence capabilities to generate true “real-time” curves of fusion indexes. Whatever, the new system developed here generated intuitive and quantitative report signals and did not require a sophisticated device or exogenous fluorophores compared with previously developed methods, such as fluorescence resonance energy transfer (FRET)^3^ or β-lactamase transfer assay.^4^

This reporting system was initially applied to compare the cell-fusion efficiency of SARS-CoV-2 Delta spike protein and its mutations. First, K417N and H655Y found in Delta or other SARS-CoV-2 spike variants were introduced into Delta spike named Delta(K417N) and Delta(H655Y). Delta(K417N) showed higher luciferase activity than Delta between 0 and 6 h, indicating that it fused faster during this time (Fig. 1f). When the H655Y mutation was added to Delta spike (Delta(H655Y)), luciferase activity significantly decreased after co-culturing the effector cells and target cells for 4–10 h, indicating decreased fusion efficiency (Fig. 1f). Second, R685G and R682W at the furin cleavage site, which led to virus growth advantage in Vero cells, were introduced into Delta spike named Delta(R685G) and Delta(R681P/R682W). Their luciferase activity and numbers of green fluorescent nuclei in red syncytia at 10 h post-cell co-culture were significantly lower than those of the other variants, including the original D614G (Fig. 1f and supplementary Fig. 3), showing substantially lower levels of cell fusion. In view of the important role of spike furin cleavage site in SARS-CoV-2 fusion, a few more mutations near the furin cleavage site found in other SARS-CoV-2 spike variants such as P681H, Q675H, and Q677H were also introduced into Delta spike (Delta(R681H), Delta(Q675H/R681P) and Delta(Q677H/R681P)) to compare their fusion efficiency. A reverse mutation Delta(R681P) was also included. The numbers of green nuclei in red syncytia and luciferase activity decreased among these mutations (supplementary Fig. 4), showing that their fusion efficiencies were slower than that of Delta. These results demonstrated that P681R mutation was more effective in accelerating cell- cell fusion than the other mutations, such as P681H, Q675H, and Q677H. All these spike proteins were expressed at comparable levels (supplementary Fig. 6).

The fusion activities of some typical spike variants of SARS-CoV-2 VOC including Omicron were then compared. After 10 h of co-culture between spike-expressing effector cells and ACE2-expressing target cells, the Delta spike variant still exhibited the highest luciferase activity, followed by Alpha, Beta, and Omicron (Fig. 1g). The same results were obtained from their numbers of green nuclei in the red syncytia region (supplementary Fig. 5). These results indicated that Delta spike possessed the highest fusion efficiency, and Omicron spike had lower cell-fusion ability than the D614G spike, consistent with a recent report.^5^ Two Omicron spike mutations Omicron(R346K) and Omicron(K679N/P681R) were constructed and the cell-fusion efficiency of them were also compared. The results showed that the fusion efficiency of these two Omicron spike mutations was improved than Omicron but not notable (Fig. 1g). All these spike proteins were expressed at comparable levels (supplementary Fig. 7).

This reporting system can also be used to evaluate the effect of fusion inhibitors. We examined Delta and Omicron spike-mediated fusion in the presence of neutralizing antibodies. REGN10933 and REGN10987, also referred to as casirivimab and imdevimab, respectively, are both RBD neutralizing antibodies that block the interaction between the spike and ACE2 proteins. Antibodies were incubated with effector cells for 30 min at 37 °C before co-cultivation. REGN10933, REGN10987, or the cocktail (REGN10933:REGN10987 = 1:1) showed a dose-dependent decrease in luciferase activity in Delta spike-mediated fusion assay (Fig. 1h), but lost the neutralizing activities in Omicron spike-mediated cell fusion (Fig. 1i).

In summary, a simple, intuitive, and robust cell fusion reporting system with three functions was established and used to analyze the fusion activity of SARS-CoV-2 spike variants. This new reporting system can also be applied in other areas, such as protein-protein interaction. Nevertheless, this study has some limitations, such as the in vitro system used in this assay differs significantly from in vivo conditions, and whether the in vitro cell-fusion activity of spike proteins is consistent with cell-fusion activity in vivo remains to be elucidated.

## Supplementary information

Supplementary Materials

## Data Availability

The data used to support the findings of this study are available from the corresponding author on reasonable request.
