# Supplementary material for: A robust reporting system for measurement of SARS-CoV-2 spike fusion efficiency
Source: Signal Transduct Target Ther. 2022 Jun 6;7:179. doi: 10.1038/s41392-022-01037-4 (PMC9169031; doi:10.1038/s41392-022-01037-4)
Supplement: Supplementary file 1 — Supplementary Materials [file 41392_2022_1037_MOESM1_ESM.docx]

Supplementary Materials for

### A robust reporting system for measurement of SARS-CoV-2 spike fusion efficiency

Cong Huang, Yang Yang, Peng Yang, Fei Wang, Xinyu Li, Xiang Song, Yiming Wang, Cuiyun Yu, Xuejun Wang, Shengqi Wang

Correspondence to: yucuiyunusc@hotmail.com; xjwang@bmi.ac.cn; sqwang@bmi.ac.cn

**This PDF file includes:**

Supplementary Materials and Methods

Supplementary Figures S1-S7

**Materials and Methods**

**Plasmid Construction and Cell Culture**

The pCMV vector used for SARS-CoV-2 spike protein expression described previously by our group was used to construct these four recombinant protein-expression plasmids ^1^, and the pLVX-IRES-tdTomato vector was used for ACE2 protein expression. The mNeonGreen protein was split into NG (1–173 amino acids) and CG (174–236 amino acids) at a point between the 173th and 174th amino acids, and NanaLuc was split into LgBiT (1–159 amino acids) and SmBiT (160–171 amino acids) at a point between the 159th and 160th amino acids. NG and SmBit were fused to different terminals of bJun named NGJS or SJNG, whereas CG and LgBit were fused to different terminals of bFos named CGFL or LFCG. HEK293T cells were cultured in Dulbecco's modified Eagle medium (DMEM; Gibco) supplemented with 10% fetal bovine serum (FBS; Gibco) and 50 IU/ml penicillin/streptomycin (Macgene) in a humidified 5% CO_2_ incubator at 37 °C.

**Construction of the Reporting System**

To detect the background-signal intensity of this system and investigate the reporter activity of different terminal connections, analysis was performed as follows. HEK293T cells seeded onto 24-well plates were transfected with four different end-fused protein plasmids pNGJS, pSJNG, pCGFL, and pLFCG individually or co-transfected with pSJNG and pCGFL, pSJNG and pLFCG, pNGJS and pLFCG, and pNGJS and pCGFL, respectively (500 ng/well). After 24 h of incubation at 37 °C, the culture medium was discarded, and the cells were resuspended in a medium containing 5% FBS and counted immediately. Then, 2 × 10^4^ cells were reseeded onto a 96-well plate. When the cells adhered onto the wall, the mNeonGreen protein expression was observed with an IX71 fluorescent microscope (Olympus), and luciferase activity was detected using the Nano-Glo Luciferase Assay Kit (Meilunbio, Dalian, China). Additionally, to test the sensitivity of this reporter system, HEK293T cells co-transfected with pNGJS and pCGFL were resuspended and serially diluted before reseeding onto a 96-well plate. The expression of mNeonGreen protein and luciferase activity were detected.

**Cell Fusion assay**

Cell-cell fusion assay, based on the new reporting system, was used to compare the cell-fusion activities of SARS-CoV-2 spike variants. HEK293T cells on a 24-well plate at a density of 70%-90% confluence was co-transfected with pNGJS and pLVX-ACE2-IRES-tdTomato plasmids (500 ng) to prepare the target cells. Similarly, HEK293T cells co-transfected with pCGFL and spike plasmids served as the effector cells, and the non-spike plasmid was co-transfected with pCGFL as the negative control. After incubation at 37 °C for 24 h, the cells in each well were detached and resuspended in fresh medium containing 5% FBS. About 2 × 10^4^ target cells were reseeded onto a 96-well plate and incubated for 5 h, subsequently, an equal amount of effector cells was added to allow cell-cell fusion. The mNeonGreen and tdTomato proteins were captured using fluorescent microscopy, and luciferase activity was detected using the Nano-Glo Luciferase Assay Kit. For real-time cell-fusion assay, the luciferase activity was detected at indicated time points. In this assay, the number of biological replicates was 3 or 4; Each experiment was repeated at least twice to ensure consistency. For fluorescence images, 3 to 5 fields of view were captured for each experiment, and a representative image was finally selected for presentation. For fusion-inhibition assay, effector cells were incubated with serial concentrations of the SARS-CoV-2 RBD-neutralizing antibodies REGN10933 or/and REGN10987 (ATMA10195Mo and ATMA10196Mo from AtaGenix company) for 30 min at 37 ℃ before co-culture. Luciferase activity was detected at 4 h post cell co-culture.

**Western Blot Assay**

The expression of spike protein plasmids was verified by western blot assay. The different spike plasmids were co-transfected into HEK293T cells with pCGFL. Transfected cells were lysed in RIPA lysis buffer mixed with protease inhibitors (Solarbio, Beijing, China), and proteins were collected by centrifugation at 4 ℃. The total protein concentration was measured by BCA method, and mixed with the loading buffer (Beyotime, Shanghai, China), and denatured at 100 ℃ for 10 min. About 20 μg of protein was separated by 10% SDS-PAGE at 150 V for 30 min and then transferred to Nitrocellulose (NC) membrane (EMD Millipore, Billerica, MA, USA). After blocking the membranes in 5% skimmed milk for 1 h, they were incubated with primary antibodies against SARS-CoV-2 S2 (Sino Biological, China) or β-actin (Solarbio, Beijing, China) for 1.5 h at room temperature. Subsequently, the membranes were incubated with HRP-labeled secondary antibodies of goat anti-rabbit or mouse (Abcam, USA) for another 1 h. Finally, protein expression was visualized using a Novex™ ECL substrate reagent kit (Thermo Fisher Scientific, Inc.) with the ECL imaging system (Tanon, Shanghai, China).

**Statistical analysis**

The data were analyzed using GraphPad Prism 8 (GraphPad Software) and presented as mean ± standard deviation (SD). Statistical significance was determined by unpaired two-tailed Student’s t test. P-values are indicated by asterisks (***P <0.001, **P <0.01, *P <0.05).

**References**

1 Yang, P. et al*.* An optimized and robust SARS-CoV-2 pseudovirus system for viral entry research. *J. Virol. Methods.* **295**, 114221 (2021).


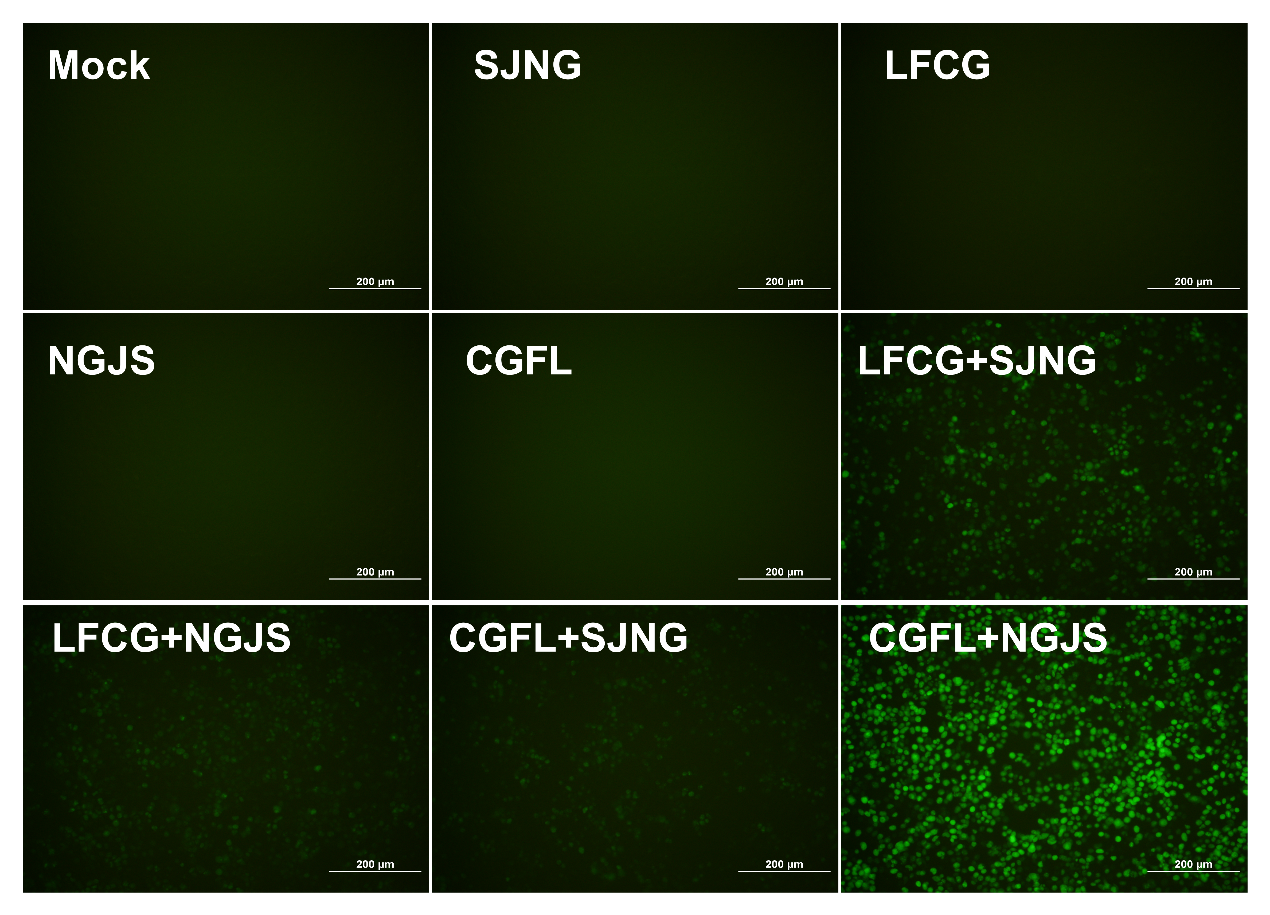


**Figure S1.** **Complementation analyses of the three-functional reporting system.** The fluorescence signal images of these four recombinant proteins after their plasmids transfected individually or co-transfected respectively into HEK293T cells for 36 h. Scale bar: 200 μm.

**
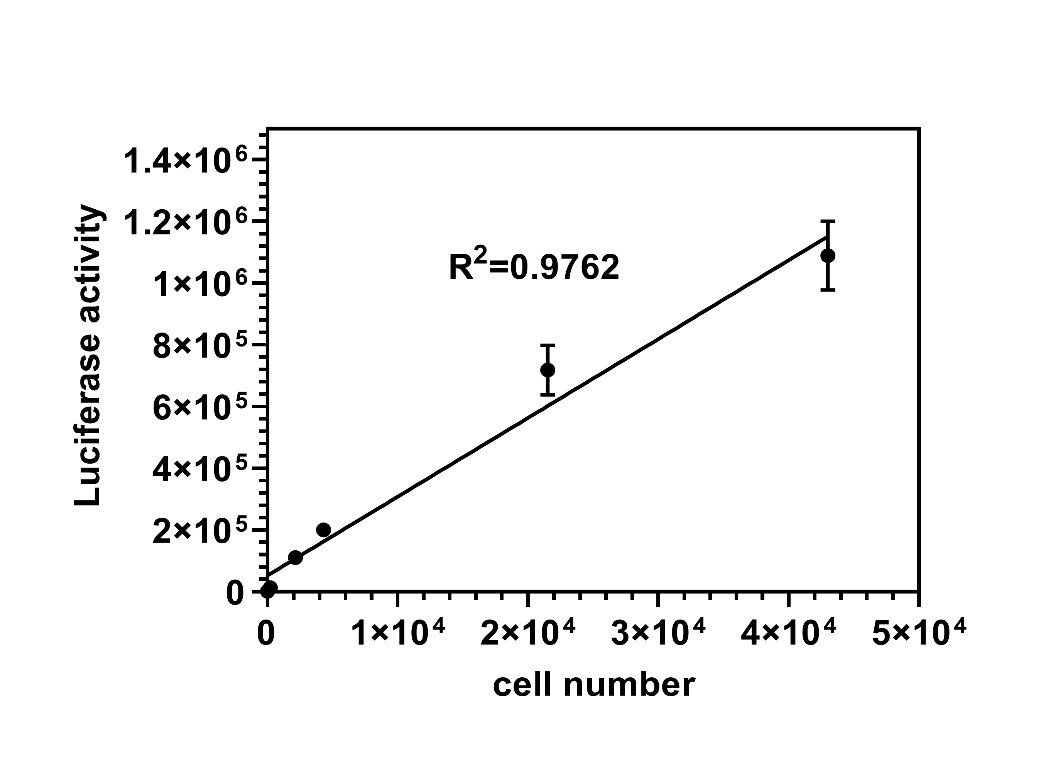
**

**Figure S2**. **Correlation between the luciferase activity and number of transfected cells**. R^2^ indicates correlation coefficiency.


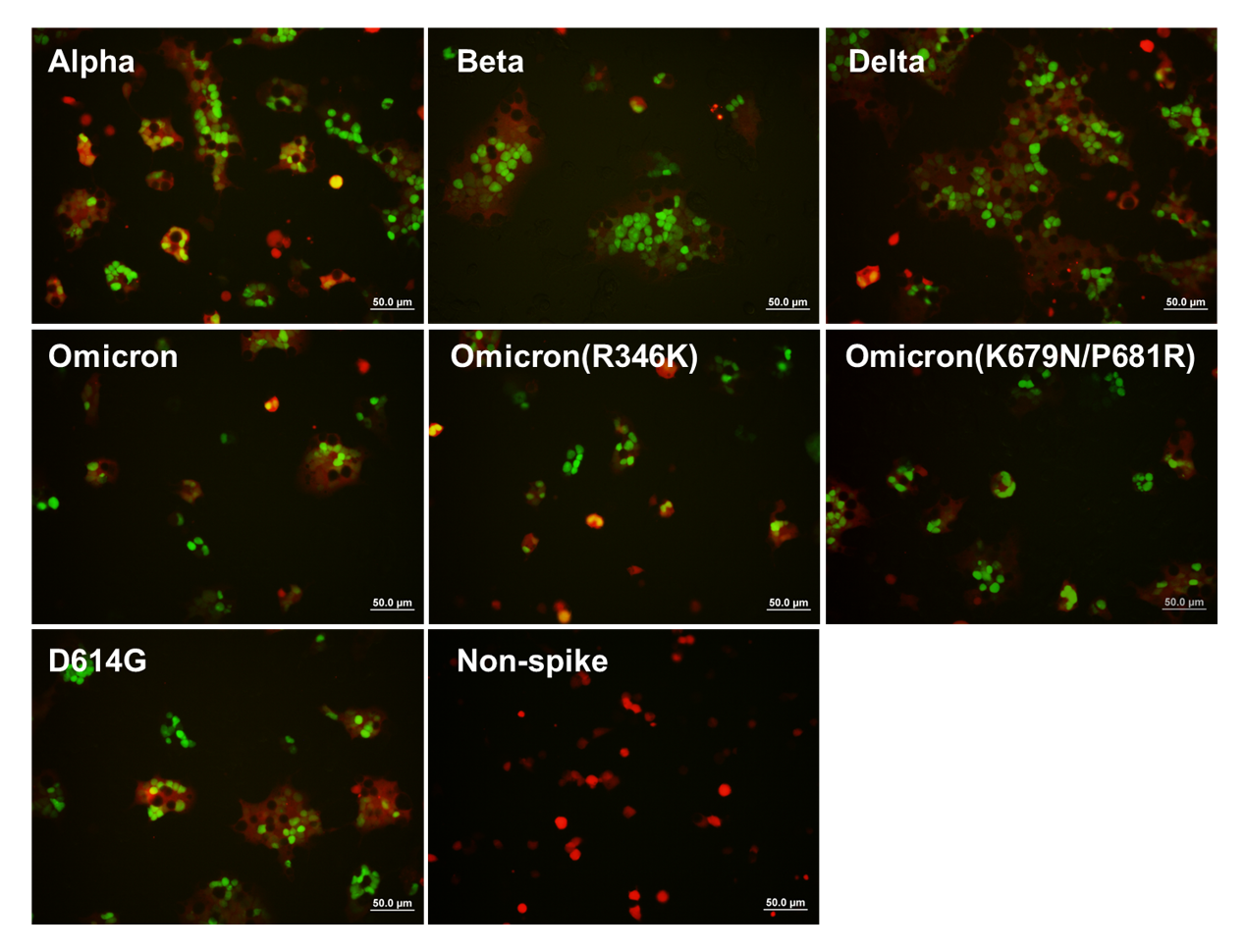


**Figure S3**. **Monitoring the cell-fusion efficiency of SARS-CoV-2 Delta spike mutants using the fluorescent microscope.** Merged images of the green fluorescence and red fluorescence at 10 h post cell co-culture. Scale bar: 50 μm.


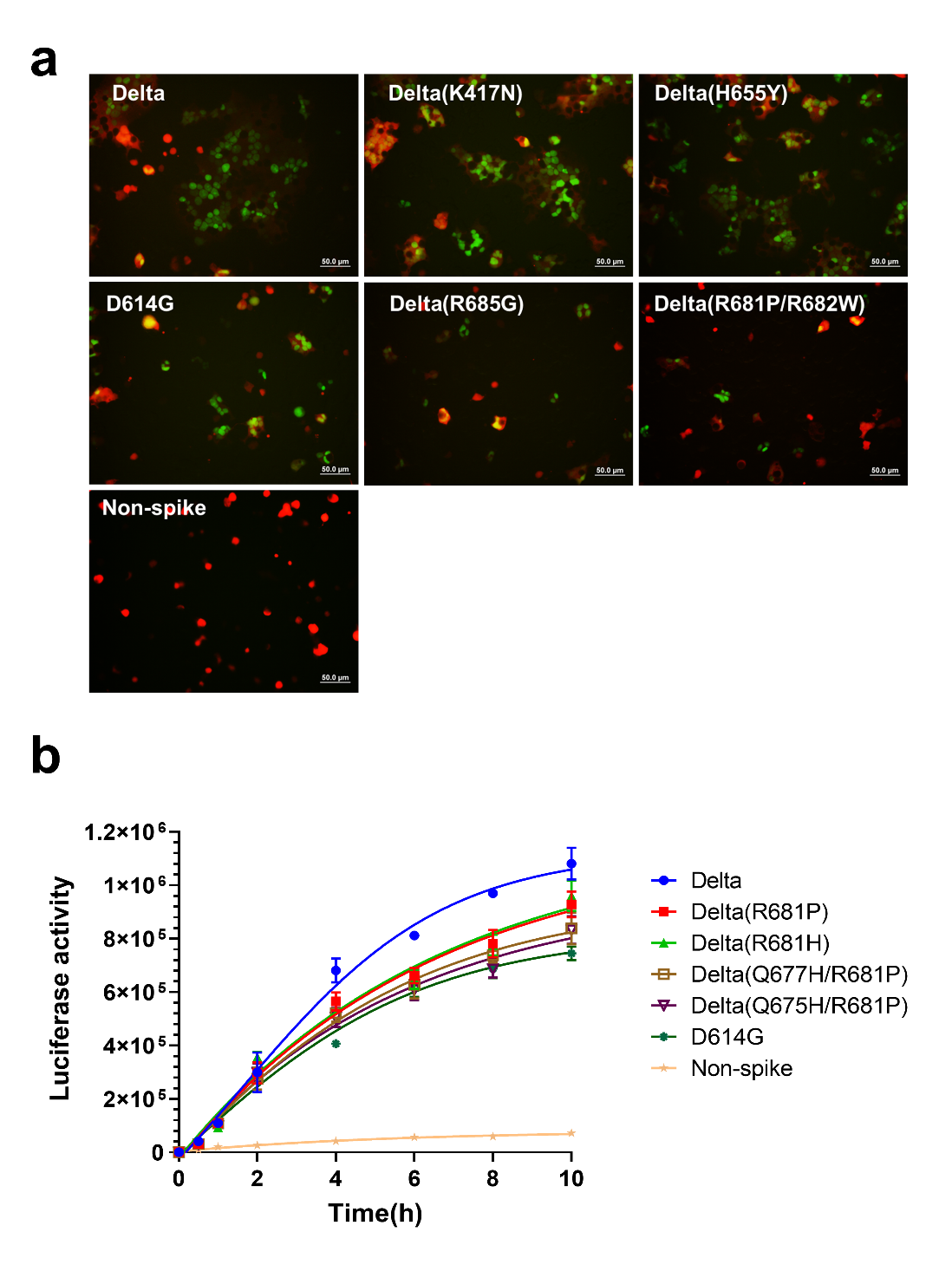


**Figure S4**. **Monitoring the cell-fusion efficiency of Delta spike proteins bearing mutations near the furin site using this reporting system. a** Merged images of the green fluorescence and red fluorescence at 10 h post cell co-culture. Scale bar: 50 μm **b** The luciferase activity at each time point was detected.


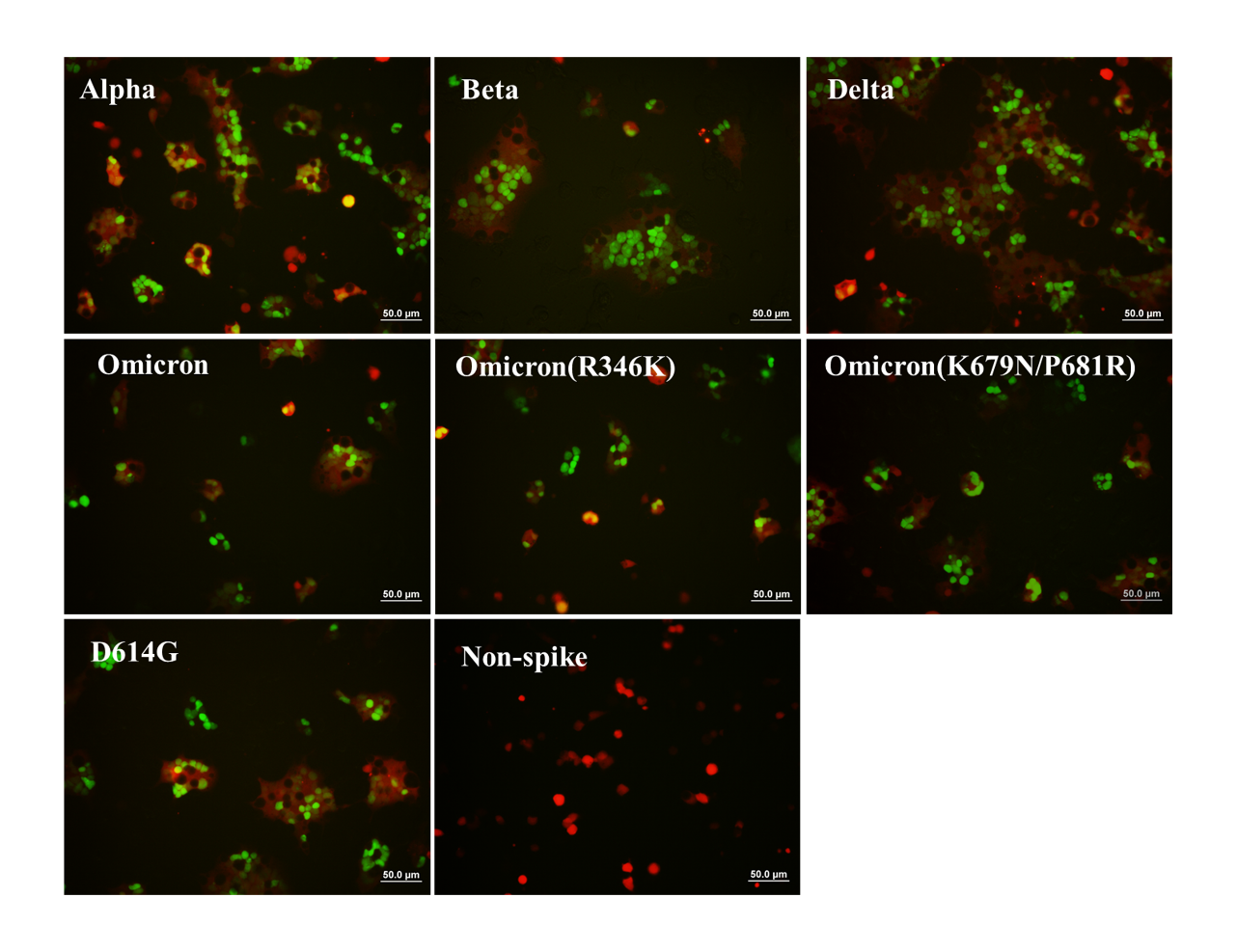


**Figure S5**. **Comparison the cell-fusion efficiency of typical SARS-CoV-2 VOC spikes and Omicron spike mutants using the fluorescent microscope**. Merged images of the green fluorescence and red fluorescence at 10 h post cell co-culture. Scale bar: 50 μm.


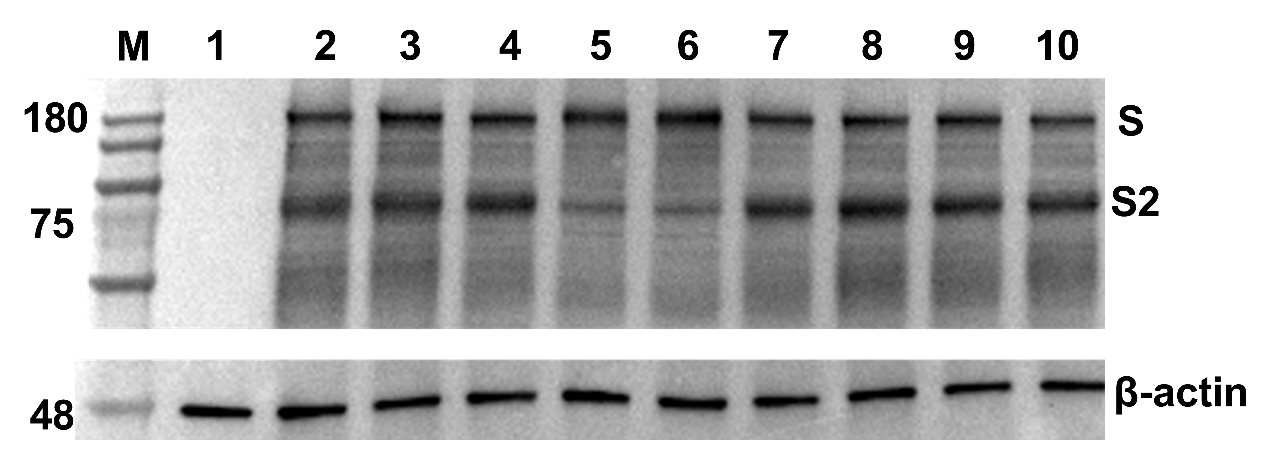


**Figure S6** **Expression of different Delta spike mutants’ plasmids in HEK293T cells** **analyzed by western blot assays.** Antibody against SARS-CoV-2 S2 subunit was used as primary antibody. Lanes 1-10 represent the negative control (non-spike), Delta, Delta(K417N), Delta(H655Y), Delta(R685G), Delta(R682W/R681P), Delta(R681P), Delta(R681H), Delta(Q677H/R681P), and Delta(Q675H/R681P), respectively.

**
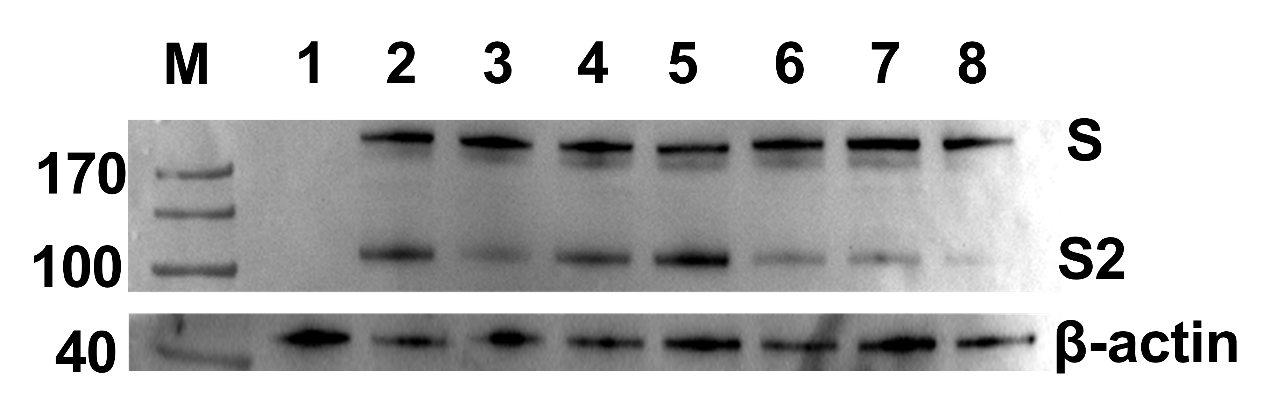
**

**Figure S7** **Expression of typical SARS-CoV-2 VOC spikes and Omicron spike mutants plasmids in HEK293T cells analyzed by western blot assays.** Antibody against SARS-CoV-2 S2 subunit was used as primary antibody. Lanes 1-8 represent the negative control (non-spike), D614G, Alpha, Beta, Delta, Omicron, Omicron(R346K), and Omicron(K679N/P681R), respectively.
